# Deep sequencing of HIV-1 reveals extensive subtype variation and drug resistance after failure of first-line antiretroviral regimens in Nigeria

**DOI:** 10.1093/jac/dkab385

**Published:** 2021-11-06

**Authors:** Kate El Bouzidi, Rawlings P. Datir, Vivian Kwaghe, Sunando Roy, Dan Frampton, Judith Breuer, Obinna Ogbanufe, Fati Murtala-Ibrahim, Man Charurat, Patrick Dakum, Caroline A. Sabin, Nicaise Ndembi, Ravindra K. Gupta

**Affiliations:** Division of Infection & Immunity, University College London, London, UK; Institute for Global Health, University College London, London, UK; Cambridge Institute of Therapeutic Immunology and Infectious Diseases, University of Cambridge, Cambridge, UK; University of Abuja Teaching Hospital, Abuja, Nigeria; Division of Infection & Immunity, University College London, London, UK; Division of Infection & Immunity, University College London, London, UK; Farr Institute of Health Informatics Research, University College London, London, UK; Division of Infection & Immunity, University College London, London, UK; U.S. Centers for Disease Control and Prevention, U.S. Embassy, Abuja, Nigeria; Institute of Human Virology Nigeria, Abuja, Nigeria; Institute of Human Virology, University of Maryland School of Medicine, Baltimore, USA; Institute of Human Virology Nigeria, Abuja, Nigeria; Institute for Global Health, University College London, London, UK; Institute of Human Virology Nigeria, Abuja, Nigeria; Africa Centres for Disease Control and Prevention, African Union Commission, Addis Ababa, Ethiopia; Cambridge Institute of Therapeutic Immunology and Infectious Diseases, University of Cambridge, Cambridge, UK; Africa Health Research Institute, Durban, South Africa

## Abstract

**Background:**

Deep sequencing could improve understanding of HIV treatment failure and viral population dynamics. However, this tool is often inaccessible in low- and middle-income countries.

**Objectives:**

To determine the genetic patterns of resistance emerging in West African HIV-1 subtypes during first-line virological failure, and the implications for future antiretroviral options.

**Patients and methods:**

Participants were selected from a Nigerian cohort of people living with HIV who had failed first-line ART and subsequently switched to second-line therapy. Whole HIV-1 genome sequences were generated from first-line virological failure samples with Illumina MiSeq. Mutations detected at ≥2% frequency were analysed and compared by subtype.

**Results:**

HIV-1 sequences were obtained from 101 participants (65% female, median age 30 years, median 32.9 months of nevirapine- or efavirenz-based ART). Thymidine analogue mutations (TAMs) were detected in 61%, other core NRTI mutations in 92% and NNRTI mutations in 99%. Minority variants (<20% frequency) comprised 18% of all mutations. K65R was more prevalent in CRF02_AG than G subtypes (33% versus 7%; *P* = 0.002), and ≥3 TAMs were more common in G than CRF02_AG (52% versus 24%; *P* = 0.004). Subtype G viruses also contained more RT cleavage site mutations. Cross-resistance to at least one of the newer NNRTIs, doravirine, etravirine or rilpivirine, was predicted in 81% of participants.

**Conclusions:**

Extensive drug resistance had accumulated in people with West African HIV-1 subtypes, prior to second-line ART. Deep sequencing significantly increased the detection of resistance-associated mutations. Caution should be used if considering newer-generation NNRTI agents in this setting.

## Introduction

ART in low- and middle-income countries is often provided without routine viral load monitoring or drug resistance testing. First-line regimens comprising two NRTIs and an NNRTI can be very successful, with one meta-analysis reporting viral suppression in over 80% of people retained in care for up to 5 years.^1^ However, suboptimal adherence and ongoing viral replication may lead to virological failure and the emergence of resistance-associated mutations in the HIV-1 RT gene.^2–5^ Infrequent monitoring may delay the recognition of virological failure and this problem may be compounded by restricted access to second-line regimens. Resistance mutations accumulate over time on failing regimens and this may lead to increased transmission of drug-resistant HIV.^6^^,^^7^

Most of the literature on HIV drug resistance is derived from partial *pol* gene Sanger sequencing technology.^3^^,^^8^^,^^9^ Studies in high-income countries with predominantly subtype B infections have shown that deep sequencing can identify more mutations than standard methods, but it is unclear whether this technology produces additional information that is clinically useful.^10^ For example, a European case–control study reported that the presence of minority drug-resistant variants prior to ART was associated with increased odds of first-line treatment failure.^11^ But this finding has not been replicated in similar analyses in sub-Saharan Africa, which found no association between baseline minority variants and first-line ART outcomes.^12–14^ Furthermore, it is not known whether minority variants present at first-line treatment failure are likely to influence the susceptibility to subsequent ART regimens.

Other parts of the genome that are not usually studied, such as the HIV-1 cleavage sites, may also affect the response to therapy.^15^ During replication, the RT heterodimer is cleaved from the gag-pol precursor protein by protease at the protease-p51 and p66-integrase cleavage sites. RT is itself cleaved into the p51 subunit and the p66 ribonuclease (RNase) H domain.^16^ These sites tend to be highly conserved, but if mutations do occur this can lead to reduced infectivity and the emergence of compensatory mutations. For example, in the p51-p66 cleavage site the F440V mutation reduces replication capacity but a compensatory mutation, T477A, restores function by allowing increased flexibility and cleavage at a different site.^17^

The greatest HIV-1 genetic diversity is seen in West and Central Africa, a region with multiple circulating clades such as subtype G, CRF02_AG and CRF06_cpx.^18^ These clades harbour natural polymorphisms, which could influence the response to treatment, and yet they are often neglected by genomics research. The AIDS Care and Treatment in Nigeria (ACTION) project was established in 2005 as a joint initiative of the Institute of Human Virology Nigeria (IHVN), the Federal Ministry of Health of Nigeria, the Institute of Human Virology of the University of Maryland and local partner facilities. The project aims to deliver an evidence-based multidisciplinary programme to improve HIV prevention and care.^19^^,^^20^ We aimed to determine the genetic patterns of resistance during first-line virological failure, prior to switch to second-line ART. Viral load testing was not part of routine care at the time of the present study. However, it was available to clinicians and could be requested if virological failure was suspected because of a deterioration, or lack of improvement, in clinical or immunological parameters. Residual plasma samples from viral load testing were stored in the IHVN biobank.

## Patients and methods

### Study population

Participants were selected from a cohort of people living with HIV who had failed first-line ART and subsequently switched to second-line therapy at the University of Abuja Teaching Hospital, Nigeria. Eligible participants were identified by searching the IHVN database and laboratory records. People aged >15 years were included if they had experienced virological failure during first-line NNRTI-based ART (HIV-1 RNA ≥1000 copies/mL, >6 months after ART initiation, confirmed by clinician-driven testing) and had a stored plasma sample available from this timepoint.

### HIV-1 whole-genome deep sequencing

Nucleic acid was extracted using the QIAamp Viral RNA Mini Kit, QIAGEN (Hilden, Germany). The cDNA was synthesized with SuperScript IV reverse transcriptase (Invitrogen, Waltham, MA, USA) and NEBNext second strand cDNA synthesis (catalogue no. E6111, New England Biolabs GmbH, Frankfurt, Germany). Sample libraries were prepared as per the SureSelect^XT^ automated target enrichment protocol (Agilent Technologies, Santa Clara, CA, USA) with in-house HIV baits.^21^ Sequencing was performed using the MiSeq platform (Illumina, San Diego, CA, USA).

### Genomic analysis

HIV subtypes were determined by the REGA subtyping tool version 3.0.^22^ All IAS–USA resistance-associated mutations detected within the viral population at ≥2% frequency were considered.^23^ The RT cleavage sites were compared with the HIV reference sequence HXB2 (GenBank accession K03455).^24^ The Stanford HIV database algorithm was used to generate two resistance reports for each sample: one based on mutations present at ≥2% frequency and one restricted to those present at ≥20% frequency.^25^ Statistical analysis was performed using Stata version 13.1 (College Station, TX, USA). The significance of subtype and other comparisons was calculated with chi-squared, Fisher’s exact, Wilcoxon signed rank or Wilcoxon Mann–Whitney tests, as appropriate.

### Ethics

Ethical approval was granted by the IHVN Institutional Review Board, the National Health Research Ethics Committee of Nigeria, the University of Maryland, Baltimore Institutional Review Board and the University College London Research Ethics Committee (14865/002).

## Results

### Study participants

Full-length HIV-1 genome sequences were generated from plasma samples from 101 participants. The participant characteristics are shown in Table 1. The median age was 30 years and two-thirds were women. ART had been started between 2005 and 2013 and participants had often been exposed to several NRTI agents over a median duration of 32.9 months. The NNRTI nevirapine was prescribed more often than efavirenz. The most common HIV-1 clades were CRF02_AG (54%) and subtype G (42%). The majority coreceptor usage was CCR5 in 66 (65%), CXCR4 in 20 (20%) and was undetermined in 15 (15%). Only 20 participants had more than one viral load performed prior to switching to second-line ART, and only 5 of those had ever had an undetectable viral load during first-line ART, therefore it was not possible to determine the duration of first-line virological failure in most cases.

**Table 1.** First-line virological failure participants’ clinical and virological characteristics

Characteristic	All participants (*n* = 101)
Sex, *n* (%)	
female	66 (65.3)
male	55 (54.5)
Age, years, median (IQR)	30 (26–37)
Baseline CD4 count^a^, cells/mm^3^, median (IQR)	129 (56–195)
ART exposure during first-line therapy, *n* (%)	
NRTI	
3TC	92 (91.1)
ZDV	75 (74.3)
TDF	45 (44.6)
FTC	42 (41.6)
d4T	34 (33.7)
ABC	3 (3.0)
NNRTI	
NVP	88 (87.1)
EFV	20 (19.8)
Time on ART at virological failure sampling, months, median (IQR)	32.9 (19.0–48.8)
HIV-1 RNA in virological failure sample, log_10_ copies/mL, median (IQR)	4.9 (4.4–5.4)
HIV-1 subtype, *n* (%)	
CRF02_AG	54 (53.5)
G	42 (41.6)
CRF06_cpx	4 (4.0)
C	1 (1.0)

3TC, lamivudine; ZDV, zidovudine; TDF, tenofovir disoproxil fumarate; FTC, emtricitabine; d4T, stavudine; ABC, abacavir; NVP, nevirapine; EFV, efavirenz. ^a^CD4, count at first-line ART initiation (eight missing).

### Drug resistance mutations

A total of 616 resistance-associated mutations were detected in the 101 samples from all participants. Minority variants, present at 2%–20% frequency in the intrahost viral population, accounted for 18.2% (112/616) of all mutations. This included 49 (8.0%) present at 2%–5% frequency and 63 (10.2%) present at 5%–20% frequency. A further 54 mutations (8.8%) were present at 20%–50% frequency, 96 (15.6%) at 50%–90% frequency and over half (354/616, 57.5%) were present at ≥90% frequency within the viral population. There were 66 mutations that were not included in the analysis as they were detected at <2% frequency and therefore could not be distinguished from low-level sequencing errors.

**Figure 1.**
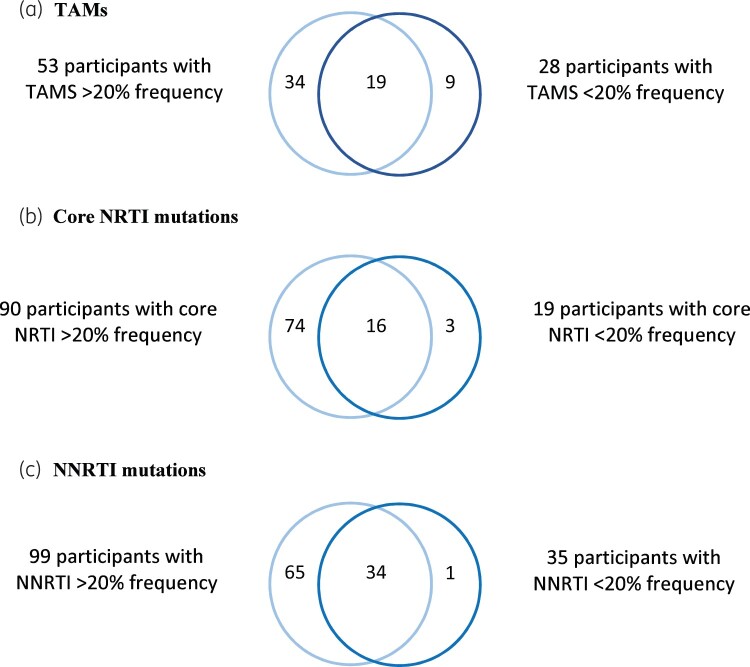
Clustering of minority variants with mutations at >20% frequency in the same class. This figure appears in colour in the online version of *JAC* and in black and white in the print version of *JAC*.

Overall, 59% of participants had minority variants. This included thymidine analogue mutation (TAM), core NRTI and NNRTI minority variants, which were detected in 28% (28/101), 19% (19/101) and 35% (35/101), respectively. Minority variants were usually detected along with other higher frequency mutations of the same class (Figure 1). However, one-third (9/28) of participants with TAM minority variants did not have any other TAM mutations at ≥20% frequency, and 16% (3/19) of those with core NRTI minority variants did not have other core NRTI mutations present at ≥20% frequency. Only one person (3%, 1/35) with an NNRTI minority variant did not have any NNRTI mutations at ≥20% frequency in the same sample. There was only one participant who had more than one class of minority variant in the absence of any resistance mutations at ≥20% frequency, and they had a core NRTI minority variant L74V at 8.6% and NNRTI minority variant E138A at 2.4%.

**Figure 2.**
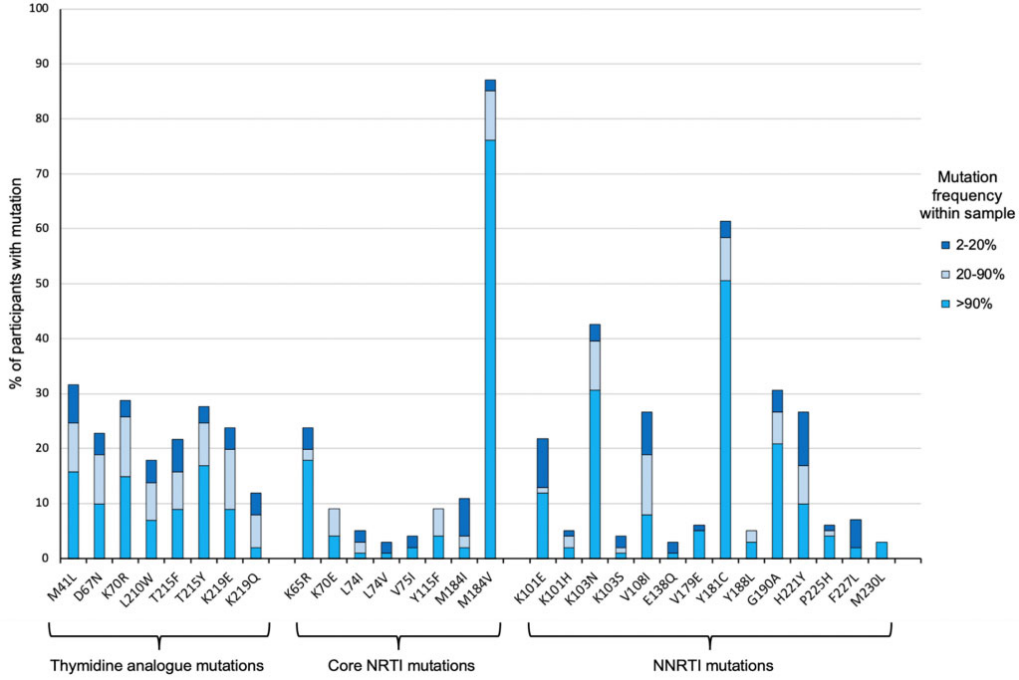
Type of RT drug resistance mutations at first-line ART failure. RT mutations shown if present in at least three participants. This figure appears in colour in the online version of *JAC* and in black and white in the print version of *JAC*.

The most common individual mutations are shown in Figure 2. M184V was present in 87% of participants (88/101) at ≥2% frequency, Y181C in 61% (62/101), K103N in 43% (41/101), M41L in 32% (32/101) and G190A in 31% (31/101). The three main types of RT mutation were all prevalent: 61% (62/101) of participants had at least one TAM, 92% (93/101) had other (non-TAM) core NRTI resistance mutations and 99% (100/101) had NNRTI resistance mutations. Over one-third of participants (37%, 37/101) had three or more TAMs. Sixteen participants had all of the TAM 1 pathway mutations (M41L, L210W and T215Y), 11 had all of the TAM 2 pathway mutations (D67N, K70R, T215F and K219Q/E) and four had the mutations for both pathways.

There was also a high prevalence of mutations among the 12 participants who were sampled less than 1 year after initiating first-line treatment: 6 (50%) had TAMs present at ≥2% frequency; 10 (83%) had core NRTI mutations (of which all 10 had M184IV and 7 also had other core NRTI mutations); and 12 (100%) had NNRTI mutations.

### Subtype variation

The K65R mutation was detected in the samples of 24% of participants (24/101), of whom 83% (20/24) had received tenofovir and 13% (3/24) had received stavudine, with two people having received both agents. All participants with K65R also had M184I/V. One-third of people with the CRF02_AG clade had K65R (33%; 18/54), compared with only 7% of people with subtype G (3/42; *P* = 0.002). Three of the four CRF06_cpx samples also had the K65R mutation. There were no significant subtype differences in NRTI exposure: 48% (26/54) of participants with CRF02_AG had received tenofovir during first-line ART, compared with 38% (16/42) of participants with subtype G (*P* = 0.33). At least one of tenofovir or stavudine had been given to 72% (39/54) of participants with CRF02_AG and to 69% (29/42) with subtype G (*P* = 0.73).

**Figure 3.**
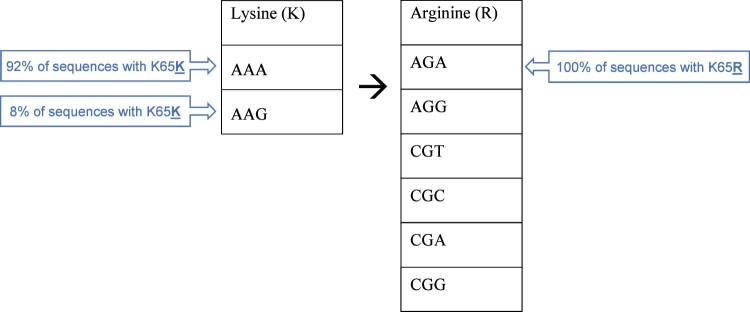
Codon usage of RT 65. This figure appears in colour in the online version of *JAC* and in black and white in the print version of *JAC*.

The codon usage in the K65 region of RT was examined to see if it varied by subtype. The K65R mutation involves a switch between the basic amino acids lysine (K), which is coded for by AAA or AAG, and arginine (R), which is coded for by AGA, AGG, CGT, CGC, CGA or CGG (Figure 3). All K65R mutations in the study sequences were AGA. Therefore, a WT codon of AAA would require only one nucleotide change to become AGA, whereas AAG would require two changes. However, the codon usage for K65K was the same among the CRF02_AG and G sequences (92% AAA and 8% AAG). One of the subtype G viruses had a K65N mutation (AAC), which causes intermediate resistance to tenofovir, abacavir, stavudine and didanosine. At the preceding codon, K64, all but three samples had the trinucleotide AAG (K64K). The exceptions to this were the subtype C sample and one subtype G, which both had the trinucleotide AAA (K64K), and one other subtype G sample, which had the trinucleotide AGG (K64R).

People with subtype G viruses were more likely to have three or more TAMs (52%; 22/42) compared with those with CRF02_AG (24%; 13/54; *P* = 0.004). There was a history of zidovudine exposure in 81% (34/42) of people with subtype G and 70% (38/54) with CRF02_AG (*P* = 0.24). Stavudine had been given to 41% (17/42) of people with subtype G and 30% (16/54) with CRF02_AG (*P* = 0.27). At least one of zidovudine or stavudine had been given to 88% (37/42) with subtype G and 78% (42/54) with CRF02_AG (*P* = 0.19). The ART duration did not differ significantly between participants with subtype G (median 36.2 months, IQR 18.2–60.7) and CRF02_AG (median 33.6 months, IQR 21.3–48.8; *P* = 0.56).

RT cleavage site mutations were found in 62% (63/101). Half (50/101) had changes in the p51-p66 cleavage site compared with the HXB2 reference amino acid sequence (GAETF/YVDGA). A phenylalanine-to-tyrosine substitution (F440Y) at the fifth codon of the cleavage site was present in 43% (43/101) of the study sequences and was more common in subtype G than CRF02_AG, at 88% and 11%, respectively (*P* < 0.001, Table 2). No sequences contained the F440V mutation. However, its compensatory mutation T477A was present in 27% (27/101) of all sequences: 51% (22/43) of those with F440Y and 9% (5/58) of those without F440Y (*P* < 0.001). A substitution in the adjacent codon, K476Q, was noted in five sequences and accompanied F440Y in three of those. Other changes in the p51-p66 cleavage site were G436K in one sequence, A437V in seven, A437T in one and E438D in three. The protease-p51 cleavage site (CTLNF/PISPI) contained mutations in nine (9%) sequences: two I851V, six P853H/S/T and two I854V. The p66-integrase cleavage site (IRKVL/FLDGI) had mutations in 17 (17%) sequences: 2 I1405V, 10 K1407R and 5 V1408I. There were no significant subtype differences observed at the protease-p51 or p66-integrase cleavage sites.

**Table 2.** RT p51-p66 RNase H cleavage site mutations

Codon	HXB2 reference	CRF02_AG^a^	G^a^	*P* value^b^
436	G	.	K_2%_	0.44
437	A	V_7%_ T_2%_	V_5%_	0.46
438	E	D_2%_	D_5%_	0.58
439	T	.	.	—
440	F	Y_11%_	Y_88%_	<0.001
441	Y	.	.	—
442	V	.	.	—
443	D	.	.	—
444	G	.	.	—
445	A	.	.	—
…				
477	T	A_4%_	A_48%_	<0.001

.indicates same as HXB2 reference sequence.

a Amino acid substitution and percentage of sequences.

b Fisher’s exact *P* value for subtype comparison.

### Impact of minority variants on predicted NRTI susceptibility

**Figure 4.**
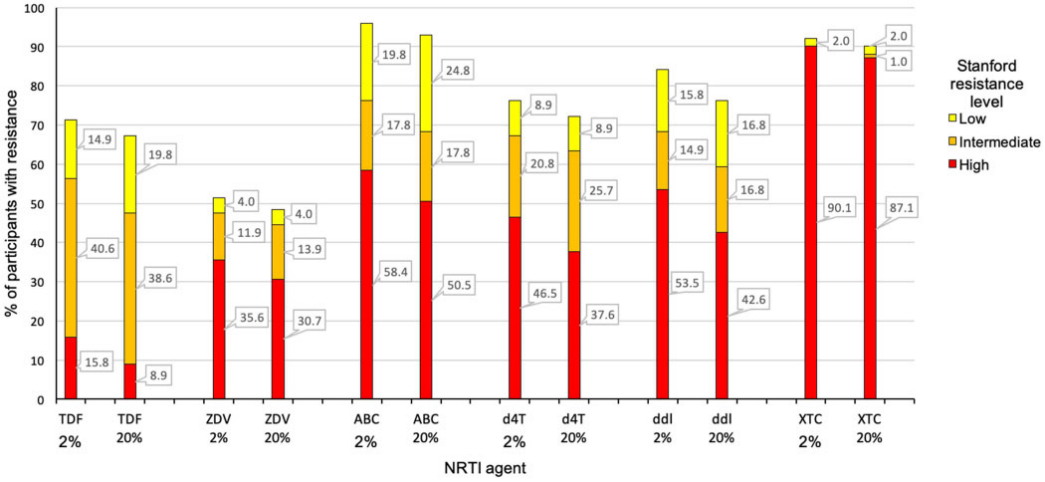
Predicted NRTI susceptibility after first-line virological failure. Proportion of participants with low-, intermediate- or high-level resistance according to the Stanford resistance algorithm at 2% and 20% mutation frequency thresholds. TDF, tenofovir disoproxil fumarate; ABC, abacavir; ZDV, zidovudine; d4T, stavudine; ddI, didanosine; XTC, emtricitabine or lamivudine. This figure appears in colour in the online version of *JAC* and in black and white in the print version of *JAC*.

The predicted susceptibility to NRTI agents is shown in Figure 4. In the case of tenofovir, using a 20% mutation frequency threshold resulted in 48% (48/101) of participants being considered to have viruses with intermediate- or high-level resistance; however, when a 2% mutation frequency threshold was used, this rose to 56% (57/101). A similar effect was seen with abacavir, with 68% (69/101) intermediate- to high-level resistance predicted when using a 20% mutation frequency threshold, but 76% (77/101) when using a 2% threshold. There was less of a difference with zidovudine: 45% (45/101) compared with 48% (48/101) with 20% and 2% mutation frequency thresholds, respectively. Most samples analysed contained high-frequency mutations that cause high-level resistance to the cytosine analogues lamivudine and emtricitabine, therefore varying the mutation frequency threshold did not make much difference for these agents. Overall, 28% of participants were predicted to have intermediate- or high-level resistance to all available NRTIs that could be given during second-line ART: tenofovir, zidovudine, abacavir, didanosine, stavudine, emtricitabine and lamivudine.

### Correlation with second-line ART outcome

All first-line virological failure sequences were predicted to be susceptible to the PIs lopinavir and atazanavir, which were prescribed for second-line therapy. However, only 11% of participants were prescribed two ‘active’ NRTI agents in their second-line regimen, to which the virus was predicted to be either susceptible or only low-level resistant. In 47% of participants, only one NRTI in their second-line regimen was predicted to be active, and in 43% no NRTIs were predicted to be active. The number of active agents in the second-line regimen did not differ significantly between participants who went on to have confirmed viral suppression and those who had virological failure during second-line ART. Of the 43 participants who received no active NRTIs, 23 (53%) had viral suppression and 20 (47%) had virological failure; of the 47 participants with one active NRTI, 19 (40%) had viral suppression and 28 (60%) had virological failure, and of the 11 participants with two active NRTIs, 4 (36%) had viral suppression and 7 (64%) had virological failure (*P* = 0.37).

### Predicted efficacy of newer-generation NNRTIs

Regardless of the mutation frequency threshold used, the Stanford algorithm predicted intermediate- to high-level resistance to nevirapine and efavirenz in >98% of participants. There was considerable cross-resistance to newer-generation NNRTI agents, to which these participants had not been exposed. Intermediate- to high-level resistance to at least one of doravirine, etravirine or rilpivirine was predicted in 81% of participants and 51% were predicted to have resistance to all three. Individually, intermediate- to high-level resistance to doravirine was predicted in 62%, to etravirine in 68% and to rilpivirine in 73%. A further 16% were predicted to have low-level resistance to doravirine, 5% to etravirine and 9% to rilpivirine.

## Discussion

First-line virological failure was associated with extensive multiclass drug resistance prior to second-line ART switch in this cohort. Deep sequencing showed that around one fifth of RT mutations were minority variants, which would not have been detected by standard resistance testing methods. The detection of highly resistant viruses less than 1 year into therapy provides further evidence that routine viral load monitoring and adherence support are likely to be crucial from the outset of treatment to preserve first-line and second-line agents and to prevent the emergence of resistance.

The K65R mutation was significantly more prevalent in CRF02_AG viruses than subtype G, which was not completely explained by differences in tenofovir exposure or treatment duration. Nor was there a difference in the codon usage at position 65 or 64 that would be expected to predispose to a lysine-to-arginine substitution, such as the run of five adenosines before the second position of codon 65, which increases the likelihood of a mutation in subtype C viruses.^26^ Participants with subtype G viruses were more likely to have TAMs and RT p51-p66 cleavage site mutations. Subtype diversity in cleavage sites has been reported in an analysis of all HIV-1 *gag* and *pol* sequences in GenBank.^16^ None of the sequences in the present study contained the F440V mutation, which has been previously shown to reduce replication capacity,^17^ but 43% had the mutation F440Y. This was often present with T477A, the compensatory mutation associated with F440V, which suggests coevolution. Further phenotypic analysis may elucidate the impact of this mutation on viral fitness and drug resistance. Subtype variation in the emergence of resistance has been previously reported, though in the case of multicentre studies it may be difficult to exclude the confounding effects of geographical and treatment centre differences.^27^^,^^28^

Most emergent resistance was already fixed in the viral population by the time of sampling, with over half of the mutations present at ≥90% frequency. Despite this, there were still many minority variants. These could represent emerging or waning resistance, or may reflect fluctuations in the frequency of mutations under changing drug pressure. Smaller studies in South Africa and Uganda also showed improved detection of drug resistance at first-line failure using deep sequencing technology.^10^^,^^29^

Lowering the mutation frequency threshold for predicting ART susceptibility resulted in more viruses being considered resistant to tenofovir and abacavir. Over two-thirds of participants had viruses predicted to be resistant to tenofovir, which is concerning as this agent is often used for second-line or third-line regimens, therefore subsequent lines of therapy could be jeopardized. Worryingly, without the benefit of genotypic resistance testing at the time of first-line virological failure, only 11% of participants were switched to second-line regimens with an NRTI backbone that was predicted to be fully active. Even if resistance testing had been available to influence the choice of second-line regimen, more than one-quarter of participants had viral genotypes that were predicted to be resistant to all available NRTI agents.

However, the NRTI resistance detected at first-line treatment failure did not appear to correlate with subsequent second-line virological response. It may be that second-line treatment success is more likely to be influenced by host factors, such as adherence. This is consistent with the finding from the EARNEST trial of second-line HIV therapy, that people with no active NRTIs in their second-line regimen did not appear to have worse virological outcomes.^30^ In fact, the number of NRTIs predicted to be active was inversely related to the likelihood of viral suppression. This may be because the presence of mutations reflects previous adherence to first-line therapy, which is then continued during second-line therapy and is associated with positive treatment outcomes.

Another issue, which may influence tenofovir susceptibility in particular, is the serendipitous effect that can occur when combinations of mutations cause lower levels of resistance than they generate individually. This is highlighted by a phenotypic analysis that showed the cytosine analogue mutation M184V mitigates against the effects of K65R, and so the overall impact on tenofovir susceptibility may not be significant when these two mutations coexist.^31^ This hypothesis was strengthened by the results of the NADIA trial, which showed that participants with K65R and M184V mutations at baseline were no more likely to fail a regimen containing tenofovir compared with zidovudine, when combined with lamivudine and either darunavir or dolutegravir.^32^ Notably, all participants in the present analysis who had the K65R mutation also had M184I/V.

An important finding was that two-thirds of participants had cross-resistance to newer-generation NNRTIs, some of which have been proposed even for people with a history of virological failure on first-generation NNRTI agents.^33^ This has implications for the future use of fixed-dose combinations such as tenofovir/lamivudine/doravirine and tenofovir/emtricitabine/rilpivirine, and for long-acting injectable combinations such as cabotegravir/rilpivirine. Many people living with HIV across the world will have received nevirapine- or efavirenz-based first-line ART, and this study adds to concerns about the impact on the efficacy of these new regimens.^34–36^ Although these people are likely to have extensive archived NNRTI resistance, this may not be detectable by standard genotyping methods by the time these regimens become widely available.

One limitation of this study is that the first-line resistance data were from people who subsequently switched to second-line ART, and therefore may not be representative of those who fail first-line ART but do not switch to second-line ART. For example, an analysis from the same study site found that one-fifth of Sanger sequences from 198 people who had failed first-line ART had no detectable drug resistance mutations.^37^ This may reflect complete non-adherence or very poor adherence, in which case a switch to second-line ART would not be common practice. On the other hand, other studies have shown that extensive resistance does occur when people remain on failing NNRTI-based regimens long term.^3^^,^^8^^,^^9^^,^^28^^,^^38^^,^^39^ Selection bias may have been introduced by clinician-driven viral load testing and the subsequent decision to switch to second-line treatment. A further limitation is the lack of baseline sequence data. It is known that some people starting ‘first-line’ ART may harbour drug-resistant virus, either from directly transmitted drug resistance or as a result of previous ART exposure in other treatment programmes or private facilities.^4^^,^^40^^,^^41^ Finally, most participants only had one viral load measurement, therefore it was not possible to infer the duration of virological failure. The duration of first-line ART (the time denominator used in this study) would overestimate the duration of unsuppressed viral replication if a participant had been fully adherent to ART initially and only developed adherence problems later in the course of treatment.

In summary, diverse West African HIV clades exhibit multiclass drug resistance with subtype variation at first-line virological failure, prior to second-line ART. The predominance of high-frequency mutations suggests that emergent resistance had become fixed in the viral population by the time of sampling. NRTI resistance was not associated with the virological response to second-line PI-containing regimens in this cohort. However, the NNRTI resistance profile suggests that newer-generation NNRTI agents are unlikely to be reliable in this setting.
